# Supplementary material for: Identification of Freezing-Responsive microRNAs and Their Targets in Chinese Jujube by Small RNA and Degradome Sequencing
Source: Int J Mol Sci. 2026 Sep 4;27(17):7912. doi: 10.3390/ijms27177912 (PMC13566890; doi:10.3390/ijms27177912)
Supplement: Supplementary file 1 [file ijms-27-07912-s001.zip › Supplementary Material S2/novel_miR_29.pdf]

Provisional ID	:	novel_miR_29
Score total	:	1.3
Score for star read(s)	:	-1.3
Score for read counts	:	0
Score for mfe	:	1
Score for randfold	:	1.6
Score for cons. seed	:	
Total read count	:	77
Mature read count	:	73
Loop read count	:	4
Star read count	:	0

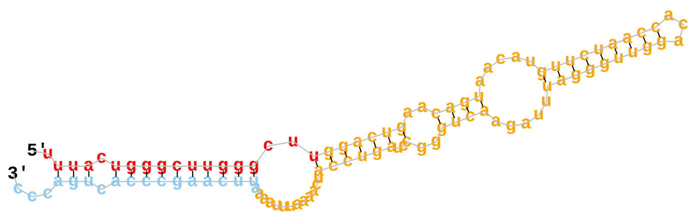

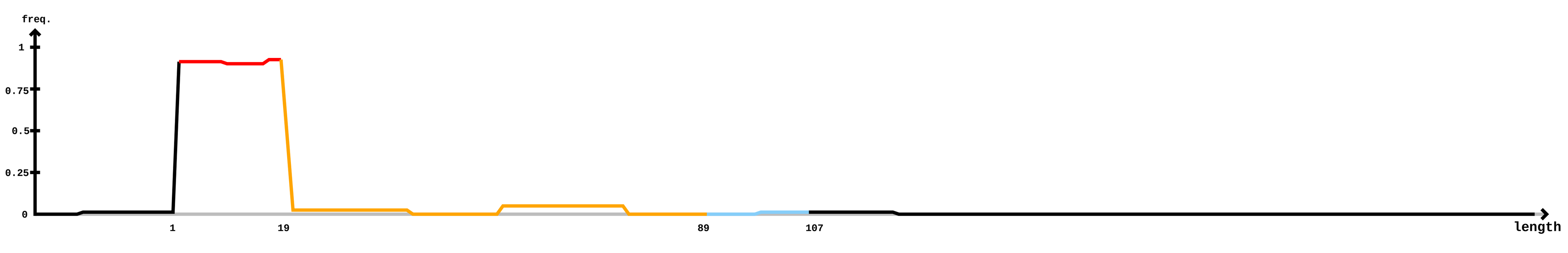

Mature Star

```
5'- cgaauuuccuugggucugggucuuuacugggcuugggcuuggacugaacaguaacauguucuaaccacagguugggauuuagaacugggccuaguccaucaaaauuuaaaauucaagcccacugacccaaaaaccuauccaaacccauuuuuccgaccuguuaacccaaauuucgacccgucuucaaccucuagccggguuuuuucgacucguuuaacugguaaacggguuuuuuagauucgguuucaau -3'   exp
    ..........(((((.((((((.....((((((((((..(((((((..((((.....(((((((((...))))))))).....))))..)..)))))).............))))))))))..))))))...)))))...(((((.((((....((((((((.(((......(((..((.....(((((......)))))....))..)))......))).))))))))....))))..)))))....   reads   mm   sample
    ......uccuugggucugggucuuuacugg............................................................................................................................................................................................................................   1   0   LT-3
    ......................uuuacugggcuugggcuu..................................................................................................................................................................................................................   36   0   LCK-1
    ......................uuuacugggcuugggcuu..................................................................................................................................................................................................................   36   0   LCK-2
    ......................uuuacugggcuugggcuuA.................................................................................................................................................................................................................   1   1   LCK-1
    .....................................cuuggacugaacaguaacauguuc.............................................................................................................................................................................................   1   0   JCK-2
    .....................................cuuggacugaacaguaacauguuc.............................................................................................................................................................................................   1   0   JCK-3
    ............................................................................auuuagaacugggccuagucc.........................................................................................................................................................   2   0   JCK-1
    ............................................................................auuuagaacugggccuagucc.........................................................................................................................................................   1   0   JCK-2
    ............................................................................auuuagaacugggccuagucc.........................................................................................................................................................   1   0   JCK-3
    .......................................................................................................................Uacugacccaaaaaccuauccaa............................................................................................................   1   1   JCK-3
```
